# Supplementary material for: Inverse Association between the Existence of CRISPR/Cas Systems with Antibiotic Resistance, Extended Spectrum β-Lactamase and Carbapenemase Production in Multidrug, Extensive Drug and Pandrug-Resistant Klebsiella pneumoniae
Source: Antibiotics (Basel). 2023 May 29;12(6):980. doi: 10.3390/antibiotics12060980 (PMC10295211; doi:10.3390/antibiotics12060980)
Supplement: Supplementary file 1 [file antibiotics-12-00980-s001.zip › Figure S1.pdf]

Figure S1. Gel images showing CRISPR and Cas elements in *Klebsiella pneumoniae* isolates.

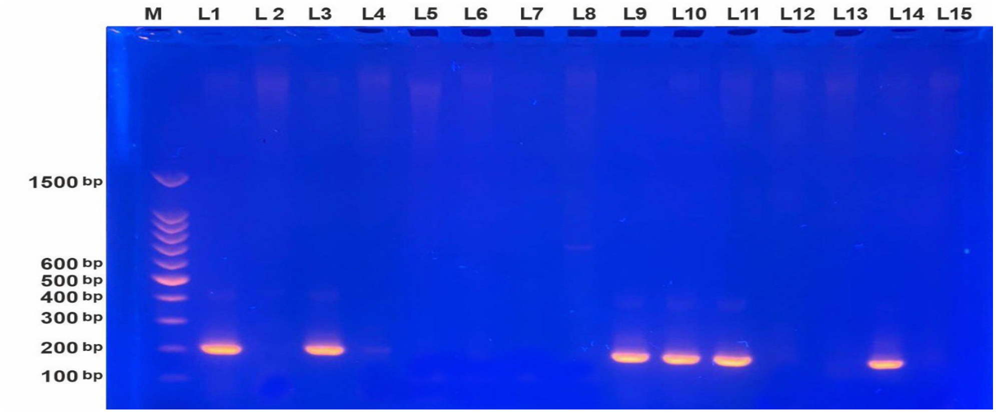

Figure S1.A: PCR product of CAS1 genes (208 bp) via PCR. M indicates the DNA marker (100 bp). The numeric characters represent the sequential number of different *Klebsiella pneumoniae* isolates.

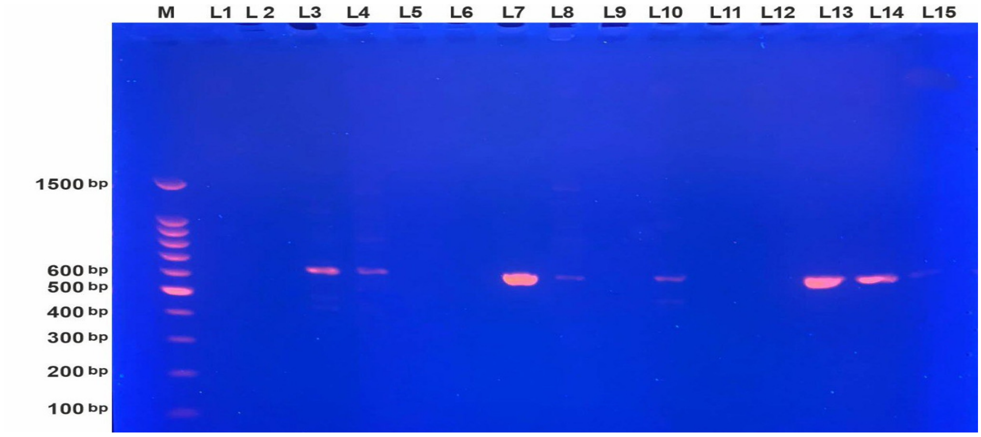

Figure S1.B: PCR product of CAS3 genes (620 bp) via PCR. M indicates the DNA marker (100 bp). The numeric characters represent the sequential number of different *Klebsiella pneumoniae* isolates.

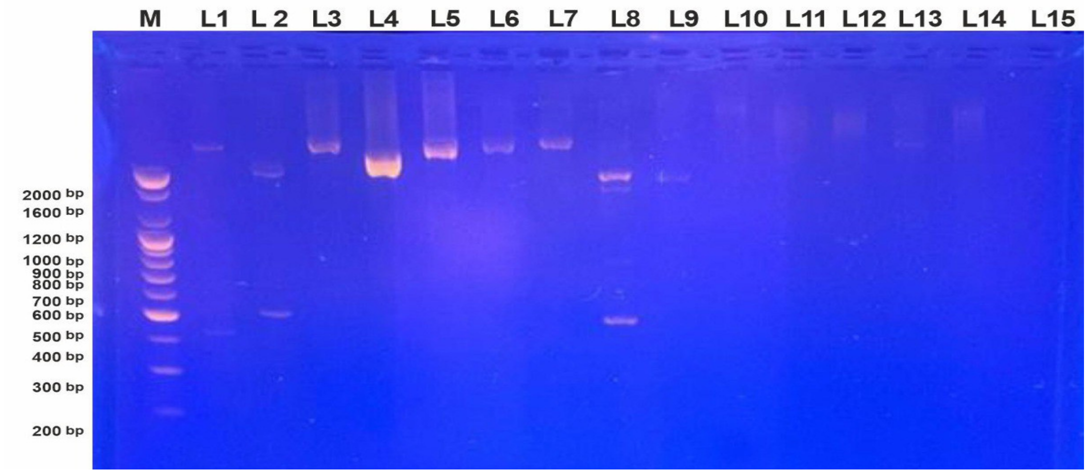

Figure S1.C: PCR product of CRISPR1 genes via PCR. M indicates the DNA marker (100 bp). The numeric characters represent the sequential number of different *Klebsiella pneumoniae* isolates that carry the CRISPR1 gene.

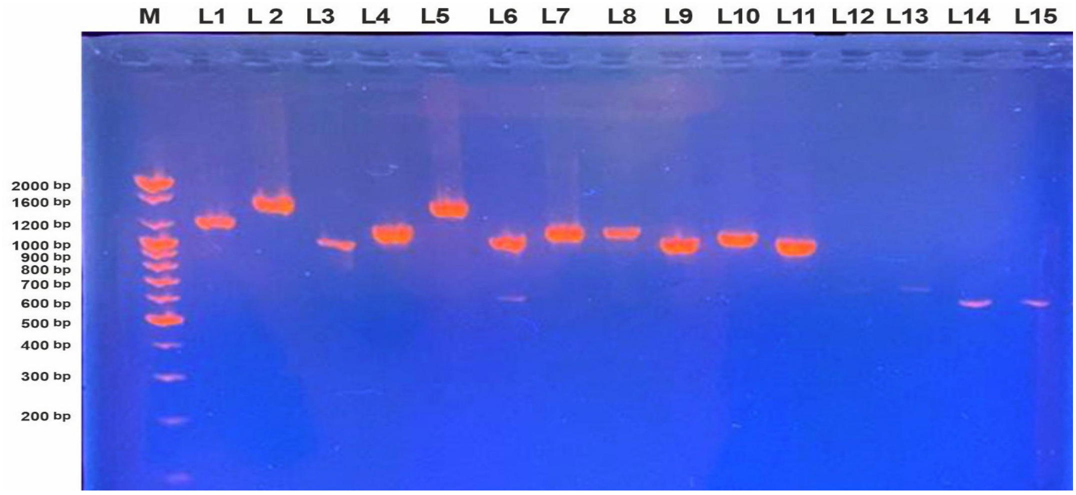

Figure S1.D: PCR product of CRISPR2 genes via PCR. M indicates the DNA marker (100 bp). The numeric characters represent the sequential number of different *Klebsiella pneumoniae* isolates that carry the CRISPR2 gene.

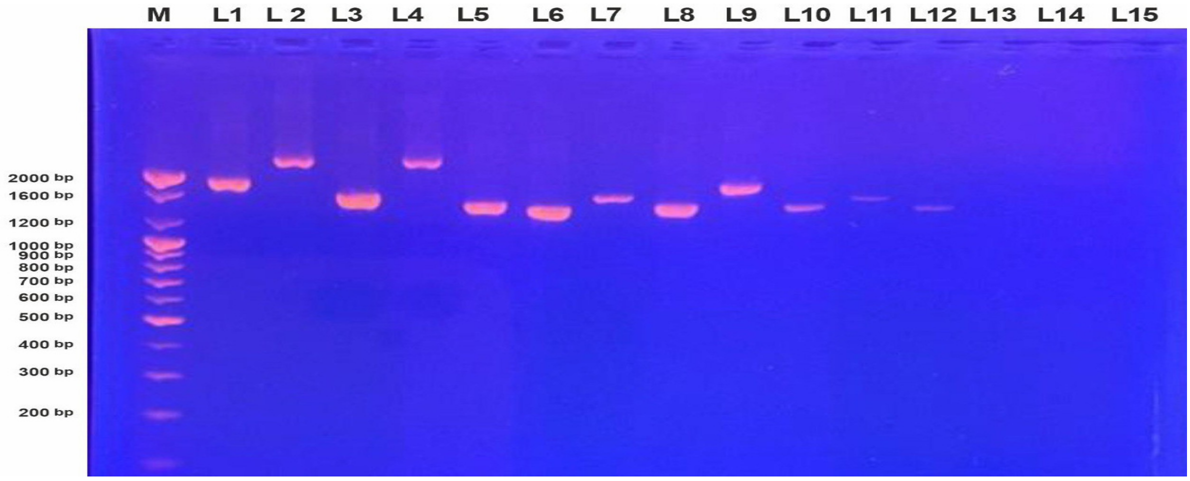

Figure S1.E: PCR product of CRISPR3 genes via PCR. M indicates the DNA marker (100 bp). The numeric characters represent the sequential number of different *Klebsiella pneumoniae* isolates that carry the CRISPR3 gene.
