# Supplementary material for: Inverse Association between the Existence of CRISPR/Cas Systems with Antibiotic Resistance, Extended Spectrum β-Lactamase and Carbapenemase Production in Multidrug, Extensive Drug and Pandrug-Resistant Klebsiella pneumoniae
Source: Antibiotics (Basel). 2023 May 29;12(6):980. doi: 10.3390/antibiotics12060980 (PMC10295211; doi:10.3390/antibiotics12060980)
Supplement: Supplementary file 1 [file antibiotics-12-00980-s001.zip › Table S1.pdf]

Table S1. The distribution of CRISPR and Cas elements and systems in *Klebsiella pneumoniae* isolates.

Table S1.A The distribution of CRISPR and Cas elements in study isolates of *Klebsiella pneumoniae* (Sensitive VS Resistant)

Sample status	Crispr1	Crispr2	Crispr3	Cas1	Cas3	P. value
Sensitive No. (%)	10(71.43)	2(14.29)	3(21.43)	10(71.43)	5(35.71)	0.013
Resistant No. (%)	17(19.77)	32(37.2)	16(18.6)	27(31.40)	37(43.02)	
Total	27 (27.0)	34 (34.0)	19 (19.0)	37 (37.0)	42 (42.0)	

N.S: No significant differences (*p* > 0.05), (*) significant differences (*p* < 0.05), (**) highly significant differences (*p* < 0.01). The Chi squared test is statistically used to compare between two proportions.

Table S1.B The distribution of CRISPR and Cas elements among resistant isolates (ESBL VS metallo β-lactamase producers)

Status	Crispr1	Crispr2	Crispr3	Cas1	Cas3	P. value
ESBL No. (%)	14(19.71)	25(35.21)	13(18.3)	23(32.39)	27(38.02)	0.806
Metallo β-lactamase No. (%)	3(20.0)	7(46.67)	3(20.0)	4(26.67)	10(66.67)	
Total	17(19.77)	32(37.21)	16(18.6)	27 (31.4)	37(43.02)	

***ESBL refers to Extended Spectrum of β-Lactamase enzyme. N.S: No significant differences (*p* > 0.05), (*) significant differences (*p* < *0.05*), (**) highly significant differences (*p* < 0.01). The Chi squared test is statistically used to compare between two proportions.**

Table S1.C The occurrence of the CRISPR /Cas system in 100 *Klebsiella pneumoniae* isolates (Sensitive VS Resistant)

Sample status	Crispr1-Cas	Crispr2-Cas	Crispr3-Cas	Crispr-Cas	P value
**Sensitive No. (%)**	6 (42.86)	1 (7.14)	1 (7.14)	8 (57.14)	0.023
**Resistant No. (%)**	7 (8.14)	23 (26.74)	16 (18.6)	24 (29.07)	0.011
**Total**	13 (13.0)	24 (24.0)	17 (17.0)	32 (32.0)	

N.S: No significant differences (p>0.05), significant differences (p < 0.05), highly significant differences (p<0.01). The Chi-squared test is statistically used to compare between two proportions.

Table S1.D The occurrence of the CRISPR /Cas system among antimicrobial-resistant isolates (ESBL VS metallo β-lactamase producer) of *Klebsiella pneumoniae*.

Sample status	Crispr1-Cas	Crispr2-Cas	Crispr3-Cas	Crispr-Cas	P. Value
**ESBL No. (%)**	6(8.45)	19(26.76)	13(18.31)	20(28.17)	0.026
**Metallo β-lactamase**	1(6.67)	4(26.67)	3(20.0)	4(26.67)	0.572
**Total**	7 (8.14)	23 (26.7)	16 (18.6)	24 (27.9)	

***ESBL refers to Extended Spectrum of β-Lactamase enzyme. N.S: No significant differences (p>0.05), significant differences (p < 0.05), highly significant differences (p < 0.01). The Chi-squared test is statistically used to compare between two proportions.**
